# ATP1A3 dysfunction causes motor hyperexcitability and afterhyperpolarization loss in a dystonia model

**DOI:** 10.1093/brain/awae373

**Published:** 2024-11-13

**Authors:** Evgeny E Akkuratov, Francesca Sorrell, Laurence D Picton, Vasco C Sousa, Martin Paucar, Daniel Jans, Lill-Britt Svensson, Maria Lindskog, Nicolas Fritz, Thomas Liebmann, Keith T Sillar, Hendrik Rosewich, Per Svenningsson, Hjalmar Brismar, Gareth B Miles, Anita Aperia

**Affiliations:** Science for Life Laboratory, Department of Women’s and Children’s Health, Karolinska Institutet, Solna 171 21, Sweden; School of Psychology and Neuroscience, University of St Andrews, St Andrews KY16 9JP, UK; Department of Neuroscience, Karolinska Institutet, Solna 171 77, Sweden; Department of Clinical Neuroscience, Karolinska Institutet, Solna 171 65, Sweden; Department of Clinical Neuroscience, Karolinska Institutet, Solna 171 65, Sweden; Science for Life Laboratory, Department of Applied Physics, KTH Royal Institute of Technology, Solna 171 21, Sweden; Science for Life Laboratory, Department of Women’s and Children’s Health, Karolinska Institutet, Solna 171 21, Sweden; Department of Medical Cell Biology, Uppsala University, Uppsala 751 23, Sweden; Department of Neurobiology, Care Sciences and Society, Karolinska Institutet, Solna 171 21, Sweden; Science for Life Laboratory, Department of Women’s and Children’s Health, Karolinska Institutet, Solna 171 21, Sweden; Science for Life Laboratory, Department of Women’s and Children’s Health, Karolinska Institutet, Solna 171 21, Sweden; School of Psychology and Neuroscience, University of St Andrews, St Andrews KY16 9JP, UK; Department of Pediatrics and Adolescent Medicine, University Medical Center Göttingen, Georg August University of Göttingen, Göttingen 37075, Germany; Clinic for Pediatrics and Adolescent Medicine, Department for Child Neurology, Developmental Neurology, General Pediatrics, Endocrinology, Diabetology, Social Pediatrics, University Hospital and Faculty of Medicine, Eberhard Karls University Tübingen, Tübingen 72076, Germany; Department of Neuroscience, Karolinska Institutet, Solna 171 77, Sweden; Science for Life Laboratory, Department of Women’s and Children’s Health, Karolinska Institutet, Solna 171 21, Sweden; Science for Life Laboratory, Department of Applied Physics, KTH Royal Institute of Technology, Solna 171 21, Sweden; School of Psychology and Neuroscience, University of St Andrews, St Andrews KY16 9JP, UK; Science for Life Laboratory, Department of Women’s and Children’s Health, Karolinska Institutet, Solna 171 21, Sweden

**Keywords:** Na^+^/K^+^-ATPase, rapid-onset dystonia-parkinsonism, spinal cord, motor control, ATP1A3 gene

## Abstract

Mutations in the gene encoding the alpha3 Na^+^/K^+^-ATPase isoform (*ATP1A3*) lead to movement disorders that manifest with dystonia, a common neurological symptom with many different origins, but for which the underlying molecular mechanisms remain poorly understood.

We have generated an *ATP1A3* mutant mouse that displays motor impairments and a hyperexcitable motor phenotype compatible with dystonia. We show that neurons harbouring this mutation are compromised in their ability to extrude raised levels of intracellular sodium, highlighting a profound deficit in neuronal sodium homeostasis. We show that the spinal motor network in *ATP1A3* mutant mice has a reduced responsiveness to activity-dependent rises in intracellular sodium and that this is accompanied by loss of the Na^+^/K^+^-ATPase-mediated afterhyperpolarization in motor neurons.

Taken together, our data support that the alpha3 Na^+^/K^+^-ATPase is important for cellular and spinal motor network homeostasis. These insights suggest that it may be useful to consider ways to compensate for this loss of a critical afterhyperpolarization-dependent control of neuronal excitability when developing future therapies for dystonia.

## Teaser

Mutations in the *ATP1A3* gene cause movement disorders characterized by dystonia. Akkuratov, Sorrell, and Picton *et al.* generate an *ATP1A3* mutant mouse, and show that the animals have dysfunctional brain and spinal cord neurons and impaired movement. These findings highlight a potential new target for dystonia treatments.

## Introduction

Na^+^/K^+^-ATPase enzymes are highly conserved and abundant proteins present in most cell types and throughout development. Na^+^/K^+^-ATPases transport three sodium ions out and two potassium ions into cells, a process consuming one molecule of ATP per pump cycle and responsible for ∼50% of brain energy consumption.^1^ Neurons express two isoforms of the Na^+^/K^+^-ATPase catalytic α-subunit: the α1 isoform (ubiquitous) and the α3 isoform (neuron-specific). High α3 expression occurs throughout the CNS, including in interneurons in the brain^2^ and spinal interneurons and motor neurons.^3,4^ The α3 isoform has a lower affinity for intracellular sodium than the α1 isoform and is responsible for the rapid extrusion of sodium that accumulates during high-frequency neuronal activity.^5^ The activity-dependent recruitment of α3-containing Na^+^/K^+^-ATPase generates a current that leads to a post-activity afterhyperpolarization (AHP) in a range of neuron subtypes throughout the brain^6-8^ and spinal cord.^4,9-11^ AHPs play an essential role in the regulation of neuronal network excitability.^6^ In contrast to most potassium ion-channel mediated AHPs, Na^+^/K^+^-ATPase-dependent AHPs exhibit a prolonged time course, tens of seconds versus milliseconds, and are therefore termed ‘ultraslow’ AHPs (usAHPs).^10^ Ouabain is a Na^+^/K^+^-ATPase-specific ligand that halts enzyme function and has higher affinity for α3- than α1-containing Na^+^/K^+^-ATPases. Since usAHPs are inhibited at low concentrations of ouabain, it has been suggested that usAHPs are mediated by α3-containing Na^+^/K^+^-ATPase.^4,7,12^

Several movement-related disorders are associated with mutations in *ATP1A3*, the gene encoding the Na^+^/K^+^-ATPase α3 subunit,^13-15^ including rapid onset dystonia parkinsonism (RDP) and alternating hemiplegia of childhood (AHC). AHC has an onset in early childhood and is associated with several severe symptoms including episodes of one-sided weakness or paralysis, dystonia, intermittent abnormal eye movements and seizures.^16,17^ RDP often has an abrupt onset of dystonia triggered by a physical or psychological stressor.^18,19^ In milder cases, symptoms may be restricted to the limbs,^20^ suggesting that *ATP1A3* mutations may cause pathophysiology of spinal motor networks, including lower motor neurons innervating the limbs. Indeed, other forms of dystonia have recently been linked to spinal cord dysfunction.^21^ However, deficits in the function of spinal motor networks have not been explored in *ATP1A3* disorders. Furthermore, the cellular mechanisms by which mutations in *ATP1A3* lead to disease remain unknown. In this study we have addressed these important gaps in our knowledge by combining molecular, cell physiological, neuronal network and whole animal studies of a missense variant, c.1838C>T (T613M), the most common *ATP1A3* mutation found in patients with RDP.^22,23^ We provide insights into the contribution of α3-containing Na^+^/K^+^-ATPases to the mechanisms controlling ionic homeostasis, neuronal excitability, locomotor network output and motor behaviour.

## Materials and methods

All animal procedures (excluding spinal cord preparations) received prior approval by the local ethical committee, Stockholms Norra Djurförsöksetiska Nämnd and were carried out following the European Communities Council Directive (86/609/EEC). Experimental procedures related to spinal cord preparations were conducted following the UK Animals (Scientific Procedures) Act 1986, with approval from the Animal Welfare Ethics Committee (AWEC) of the University of St Andrews in line with UK Home Office regulations. Full details of the methods are provided in the Supplementary material.

## Results

**Figure 1:**
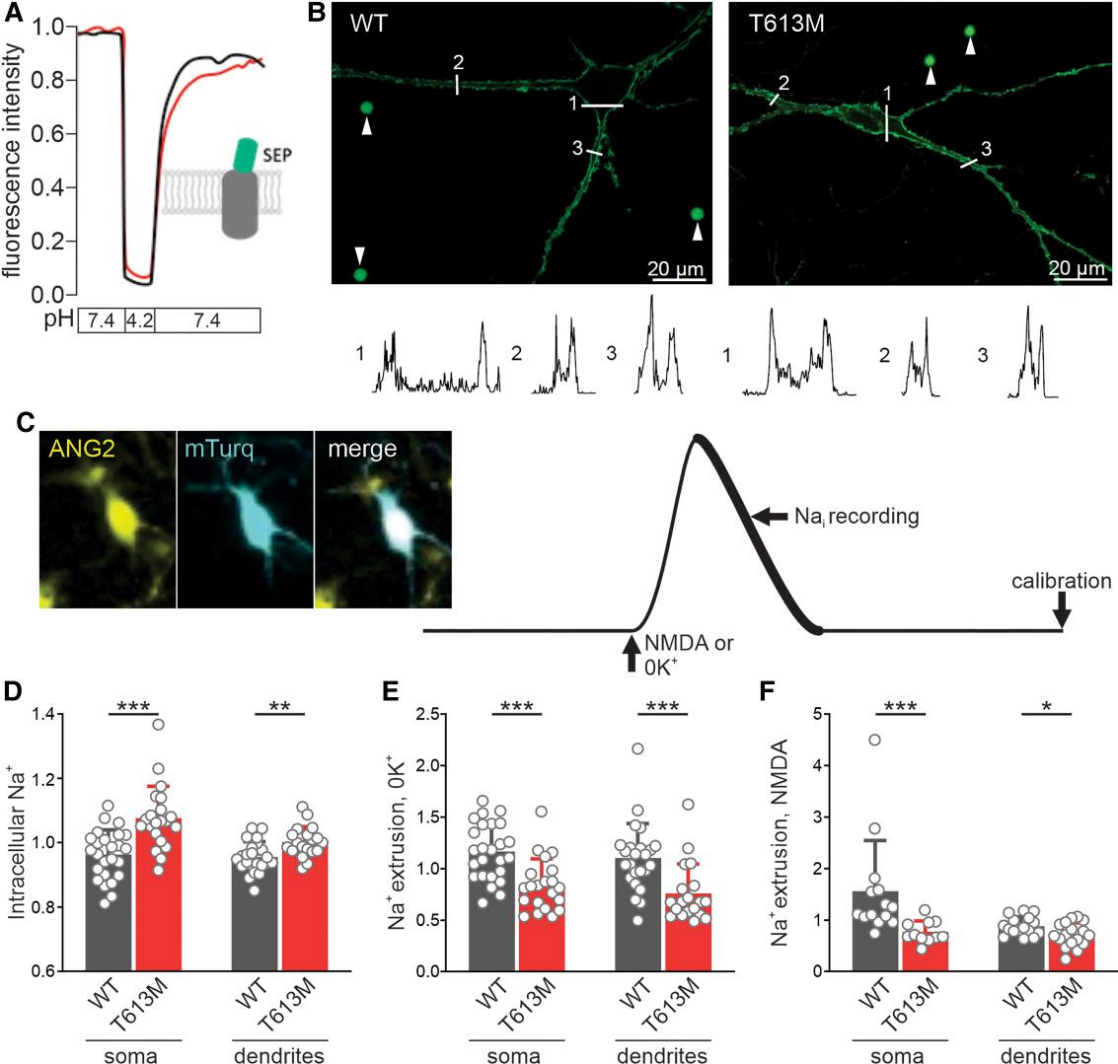
**Impaired intracellular sodium homeostasis in T613M mutant neurons.** (**A**) Similar fluorescence levels of the pH-sensitive Superecliptic pHluorin (SEP) probe tagged to the α3 Na^+^/K^+^-ATPase subunit were found in wild-type (WT, black) and T613M mutant (red) neurons. Fluorescence transiently decreased when extracellular pH was reduced to 4.0 for both WT and mutant cells. (**B**) Representative neurons expressing WT or T613M α3 tagged SEP. White arrows indicate fluorescent beads used for calibration. Traces below show fluorescence intensity profiles in regions marked by white lines. (**C**) Representative image of neuron expressing mTurquoise following loading with a Na-sensitive dye (ANG2). Schematic on *right* shows protocol for measuring sodium extrusion rate. (**D**) Basal intracellular sodium levels were significantly higher in T613M mutant cells compared with WT (soma: *n* = 25 WT, *n* = 21 T613M; dendrites: *n* = 24 WT, *n* = 19 T613M; Mann–Whitney rank test). (**E**) Sodium extrusion rate was significantly reduced in T613M mutant cells compared with WT following exposure to zero K^+^ solution (soma: *n* = 25 WT, *n* = 21 T613M; dendrites: *n* = 24 WT, *n* = 19 T613M; Mann–Whitney rank test). (**F**) Sodium extrusion rate was significantly reduced in T613M mutant cells compared with WT following exposure to NMDA (soma: *n* = 14 WT, *n* = 11 T613M; dendrites: *n* = 16 WT, *n* = 17 T613M; Mann–Whitney rank test). *P < 0.05, **P < 0.01, ***P < 0.001.

We first compared the capacity of cells expressing wild-type (WT) and mutant *ATP1A3* to manage increases in intracellular sodium. T613M and WT *ATP1A3* were expressed in cultured murine hippocampal neurons. To verify the plasma membrane location, the α3 Na^+^/K^+^-ATPase subunit was tagged in the extracellular domain between transmembrane domains 3 and 4 with a pH-sensitive fluorescent protein (Fig. 1A and B). Similar levels of extracellular pH-sensitive fluorescence were found in T613M and WT cells. Next, T613M and WT neurons were loaded with the sodium-sensitive dye Asante Natrium Green 2 (ANG2; Fig. 1C) and the fluorescence level was assessed at both the soma and at the level of neuronal extensions, the vast majority likely to be dendrites. Basal intracellular sodium concentration was significantly higher in both the soma and dendrites of T613M neurons when compared with WT neurons (Fig. 1D). To examine the capacity of the neurons to rapidly normalize an increase in intracellular sodium, WT and T613M neurons were briefly exposed to a potassium-free solution or to NMDA (Fig. 1C, E and F). The sodium extrusion rate was significantly slower in both the soma and dendrites of T613M neurons compared with WT neurons following exposure to both potassium-free solution (Fig. 1E) and to NMDA (Fig. 1F). In addition, whole-cell patch clamp recordings demonstrated greater excitability of T613M neurons compared with WT neurons, as evidenced by depolarized resting membrane potentials and a greater frequency of spontaneous action potentials (Supplementary Fig. 1). We next examined the effects of T613M on sodium handling in another cell type, striatal neurons. Similar differences in sodium extrusion rate were observed when T613M was expressed in cultured striatal neurons (Supplementary Fig. 2). These results highlight a profound deficit in sodium extrusion capacity in neurons expressing the T613M mutation. Indications of a reduced capacity to restore sodium were also observed in neurons expressing three other *ATP1A3* mutations (I274T, F780L, D801Y) identified in patients with RDP (Supplementary Fig. 3).

**Figure 2:**
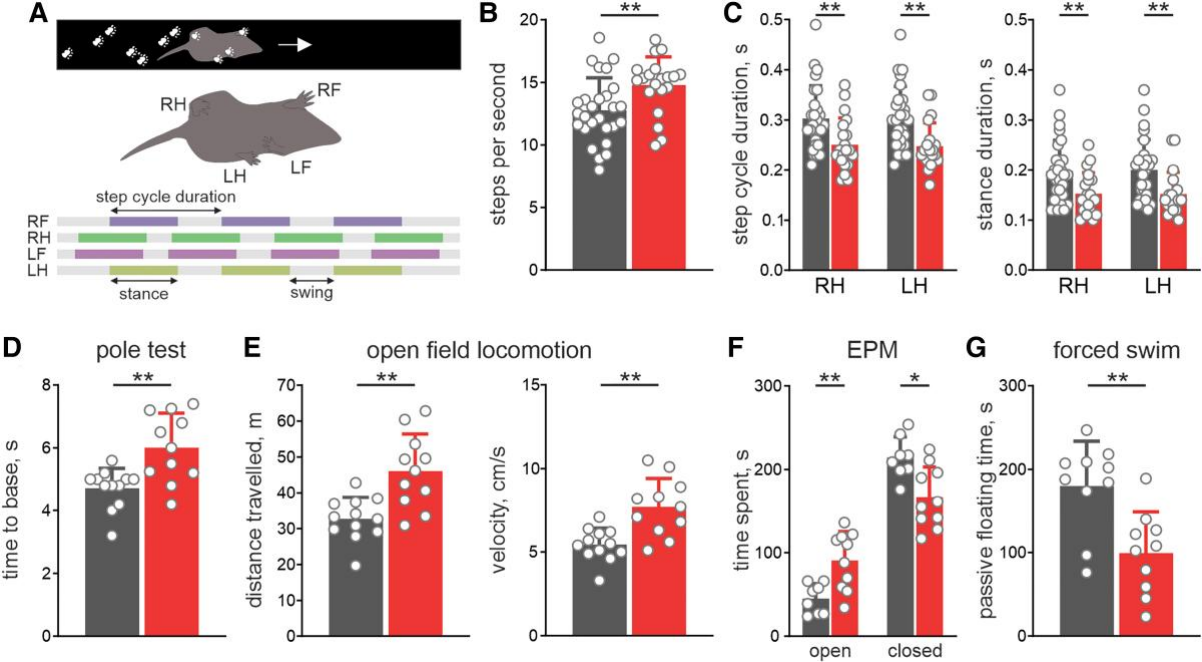
**Motor hyperexcitability behavioural phenotype in T613M mice.** (**A**) Schematic showing kinematic parameters analysed in the CatWalk experiments. (**B**) T613M mice (red, *n* = 20) showed a higher frequency of stepping compared with wild-type (WT) mice (black, *n* = 27, Mann–Whitney Rank test). (**C**) T613M mice (*n* = 20) had a shorter hindlimb step cycle duration (*left*) involving a reduced stance duration (*right*) compared with WT mice (*n* = 27, Mann–Whitney Rank test). (**D**) T613M mice (*n* = 11) showed deficits in the pole test compared with WT (*n* = 12). (**E**) Overall distance travelled (*left*) and velocity of locomotion (*right*) in the open field test were higher in T613M mice (*n* = 11) compared with WT (*n* = 12, Mann–Whitney Rank test). (**F**) T613M mice (*n* = 10) spent more time in the open arm and less in the closed arm in an elevated plus-maze (EPM) compared with WT (*n* = 8, Mann–Whitney Rank test). (**G**) T613M mice (*n* = 10) spent less time passively floating in a forced-swim test compared with WT (*n* = 10, Mann–Whitney Rank test). *P < 0.05, **P < 0.01. F = forelimb; H = hindlimb; L = left; R = right.

We next generated a knock-in mouse model of the T613M mutation to determine if the sodium extrusion deficits observed in cultured neurons translate to a behavioural phenotype. The human RDP phenotype often involves an abrupt onset of symptoms, which was only rarely observed in T613M mice and applications of physiological triggers (immobilization, cold water stress and alcohol injection) did not lead to progression of RDP-related symptoms. We did, however, observe three animals that spontaneously developed pronounced mobility deficits, including tremors (Supplementary Video 1). Additionally, analysis of motor behaviours revealed consistent and significant effects in T613M animals. A CatWalk gait analysis of the locomotor performance showed that T613M mice walked with a higher frequency of stepping (Fig. 2A and B), shorter step cycle duration (Fig. 2C) and a reduction in the stance phase duration across all limbs (Fig. 2C and Supplementary Fig. 4). The altered motor coordination, hyperactivity and altered gait pattern (Supplementary Videos 2 and 3) are consistent with functional alterations in the central pattern generator (CPG) networks of the spinal cord that generate locomotor output. In the pole test, T613M mice showed impaired motor coordination and took significantly longer to descend to the base (Fig. 2D). However, the increased latency in the pole test was not due to global hypo-locomotion. In fact, T613M mice showed hyperlocomotion with increased speed in the open field test (Fig. 2E). The distance travelled by T613M mice was approximately 50% greater than in WT mice (Fig. 2E). Furthermore, T613M mice spent more time in the open arms in the elevated plus maze (Fig. 2F) and reduced time passively floating in the forced swim test (Fig. 2G). However, the overall hyper-locomotion phenotype in these mice may have influenced the results of these motor-based tests for anxiety- and depression-like behaviours, respectively. Taken together, the major behavioural deficits observed in the T613M mice relate to motor coordination and locomotor rhythm generation and are compatible with the findings in several other mouse models of dystonia.^24^

**Figure 3:**
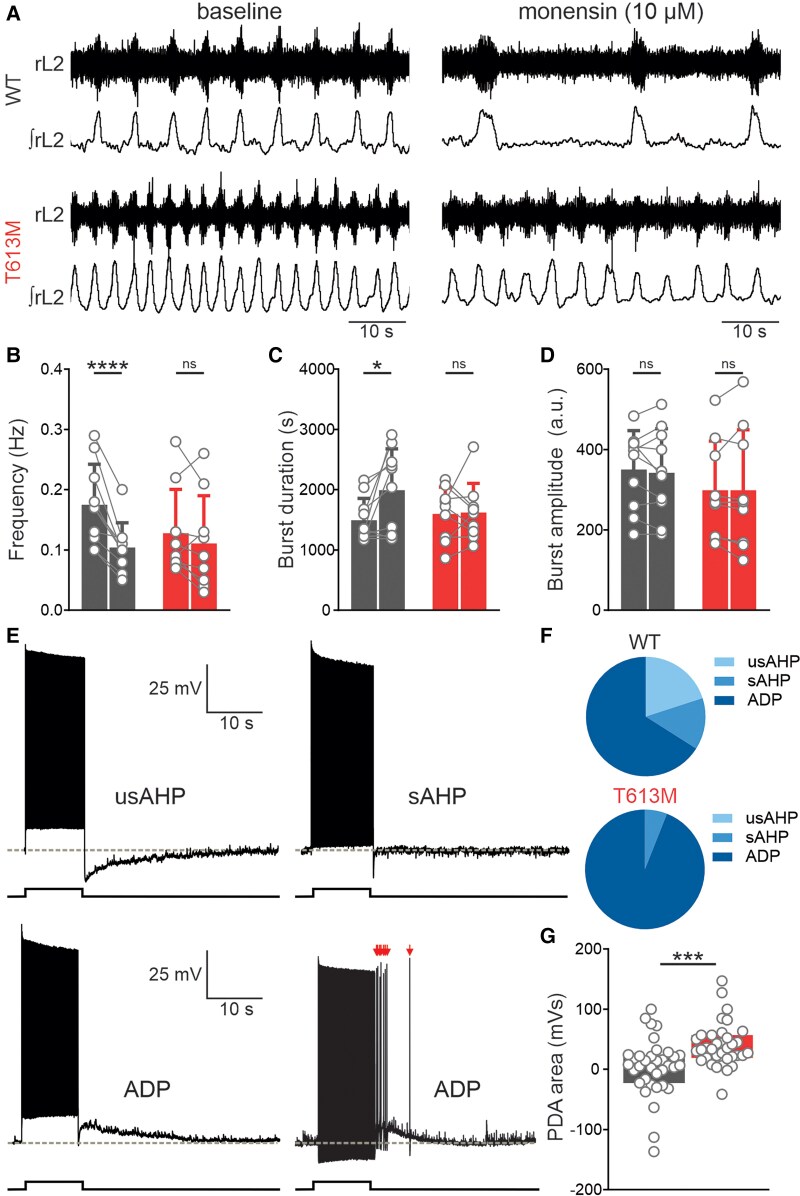
**Loss of activity-dependent homeostasis in the spinal locomotor network of T613M mice.** (**A**) Raw ventral root recordings with rectified/integrated traces showing fictive locomotion in wild-type (WT, *top*) and T613M (*bottom*) spinal cords before and after application of monensin. (**B**) Monensin significantly reduced locomotor frequency in WT spinal cords (black, *n* = 10, paired *t*-test) but not in T613M spinal cords (red, *n* = 9, paired *t*-test) within 5 min of drug application. (**C**) Monensin increased locomotor burst duration in WT spinal cords (*n* = 10, paired *t*-test) but not in T613M spinal cords (*n* = 9, paired *t*-test). (**D**) Burst amplitude was not affected by monensin in either WT (*n* = 10, paired *t*-test) or T613M spinal cords (*n* = 9, paired *t*-test). (**E**) Raw recordings of motor neurons highlighting post-spiking responses. Red arrows indicate spiking during afterdepolarization (ADP) following the offset of the stimulus. (**F**) Proportion of post-spiking responses. T613M mutant mouse motor neurons do not display ultra-slow afterhyperpolarizations (usAHPs) and show an increase in the proportion of ADP responses. (**G**) Post-discharge area (PDA) was significantly more depolarized in T613M motor neurons (*n* = 31) compared with WT (*n* = 35, unpaired *t*-test). *P < 0.05, ***P < 0.001, ****P < 0.0001. ns = not significant.

Previous studies support contributions of the basal ganglia and cerebellum towards the pathophysiology of RDP.^26-29^ However, whether motor circuits within the spinal cord may contribute has not been tested. Therefore, we next tested whether the T613M mutation affected the ability of neurons in the spinal locomotor network to respond to activity-dependent increases in intracellular sodium. We used *in vitro* isolated spinal cord preparations obtained from neonatal T613M and WT mice, in which locomotor-related motor output was induced pharmacologically and recorded from ventral roots. When WT preparations were exposed to monensin, a sodium ionophore that equilibrates intra- and extracellular sodium concentration,^25^ the frequency of locomotor-related output was reduced and the burst duration increased within 5 min of drug application (Fig. 3A–D). This effect is consistent with previous work demonstrating a negative feedback role for the Na^+^/K^+^-ATPase within spinal motor networks and is consistent with the anticipated effects of raising intracellular sodium on Na^+^/K^+^-ATPase-mediated currents.^11^ In contrast, at the same time point following exposure to monensin, no significant changes were observed in the locomotor-related output recorded from T613M spinal cords (Fig. 3A–D). This finding highlights a reduced ability of the spinal motor network in T613M mutant mice to compensate homeostatically for activity-dependent rises in intracellular sodium levels, consistent with the effects on cultured neurons carrying the T613M mutation.

Next, we studied the cellular mechanisms underlying the reduced locomotor network response to increases in intracellular sodium. Previous studies, across a range of species,^9-11^ have revealed long duration, Na^+^/K^+^-ATPase-dependent usAHPs in motor network neurons. These usAHPs are thought to provide neuroprotective, activity-dependent inhibition by reducing the excitability of motor networks following periods of intense activity. Since the sodium excretory capacity following NMDA exposure is lower in neurons expressing T613M than in WT neurons (Fig. 1), we next tested if the capacity to produce usAHPs might be perturbed in the mutant mice. We found that, unlike in control motor neurons, none of the recorded T613M-affected motor neurons (*n* = 0/100) exhibited usAHPs and that they were significantly more likely to remain depolarized following periods of intense activity compared with WT motor neurons (Fig. 3E–G). This depolarization could, in turn, lead to sustained periods of action potential firing following the offset of depolarizing input (see example in Fig. 3E). Most other intrinsic properties of T613M-affected motor neurons remained unaltered (Supplementary Table 1). These results provide the first direct evidence that the α3 subunit plays a pivotal role in the activity-dependent usAHP generated by increased Na^+^/K^+^-ATPase activity, and that this neuroprotective mechanism is abolished in motor neurons in our T613M mutant mouse model of dystonia.

## Discussion

Taken together, we demonstrate at the cellular, network and whole-animal level that an *ATP1A3* mutation leading to RDP results in profound disruption of normal neuronal function with impairment of locomotion. Our study highlights a fundamental role for the α3-containing Na^+^/K^+^-ATPase in regulating neuronal firing and network excitability through an activity-dependent usAHP mechanism that allows neurons to self-regulate their firing and homeostatically stabilize their activity following prolonged or intense activation.

Previous studies have strongly implicated the cerebellum and basal ganglia in models of *ATP1A3*-related disorders.^26-29^ By using an isolated spinal cord preparation, we provide novel evidence for an additional role of spinal motor networks. However, since *ATP1A3* is expressed by neurons throughout the nervous system, it is likely that the Na^+^/K^+^-ATPase acts as a regulator of excitability, not only in motor neurons and motor networks, but also in other networks throughout the CNS. As such, future investigation into the contribution of the activity-dependent usAHP throughout the rostrocaudal neuraxis, particularly the cerebellum and basal ganglia, is likely to aid in the development of a complete understanding of the pathophysiology of this model. Furthermore, future strategies that aim to selectively disrupt α3-containing sodium pumps in the spinal cord will be informative in assessing the relative contributions of CPG dysfunction towards RDP symptoms.

Dystonia is a common symptom in a large range of movement disorders, but little is known about the molecular mechanisms underlying dystonic symptoms and no specific therapies exist. Our data provide strong evidence that loss of a sodium-dependent AHP leads to aberrant neural activity in a novel mouse model of dystonia. This key protective role for an AHP is consistent with other work reporting that a mutation in the *HPCA* gene encoding a calcium sensor protein, which has been identified in a group of patients with severe dystonia symptoms, results in loss of a calcium-dependent slow AHP.^30^ Considering our evidence for a relationship between dystonia and loss of Na^+^/K^+^-ATPase-dependent function, we propose that efforts to develop new therapies for patients suffering from dystonia should focus on compensating for loss of function or strengthen the activity of proteins that are of crucial importance for AHP processes. Given the current lack of clinically appropriate pharmacological agents with such actions, we believe that drug development focused on the Na+/K^+^-ATPase and the AHP may prove fruitful. It may also be useful to consider other ways of addressing sodium handling, for example, by regularly monitoring serum sodium concentration and considering dietary sodium intake. In addition, it will be important to continue the development of gene therapies for devastating movement disorders, including those involving dystonia.

## Supplementary Material

awae373_Supplementary_Data

## Data Availability

Data used in this study are available within the article and its Supplementary material. Additional data underpinning this publication can be accessed at https://doi.org/10.17630/767a6828-a745-4c94-8ffd-a7734ab6ea91.
