# Supplementary material for: ATP1A3 dysfunction causes motor hyperexcitability and afterhyperpolarization loss in a dystonia model
Source: Brain. 2024 Nov 13;148(4):1099–105. doi: 10.1093/brain/awae373 (PMC11967811; doi:10.1093/brain/awae373)
Supplement: awae373_Supplementary_Data [file awae373_supplementary_data.zip › brain-2024-01183-File005.pdf]

Supplementary material

Supplementary methods

Primary hippocampal cell culture. Hippocampal neurons were prepared from embryonic day 18 Sprague Dawley rat embryos of both sexes. Hippocampus was removed, washed in Hank's balanced salt solution (HBSS) containing 20 mM HEPES then incubated for 10 min at 37°C in HBSS with 20 mM HEPES and 0.25% trypsin and dissociated in minimum essential media (MEM) by pipetting using a fire-polished Pasteur pipette. Cells were plated at a density of 4 * $10^4/cm^2$ for imaging or 10 * $10^4/cm^2$ for immunocytochemistry on glass coverslips previously coated overnight with poly-ornithine (80 µg/ml) in MEM containing 10% horse serum, 2 mM L-glutamine, and 1 mM sodium pyruvate for three hours. Then the medium was replaced with Neurobasal medium containing 2% B27, 0.5 mM L-glutamine, and 1% penicillin/streptomycin. Half the medium volume was replaced with Neurobasal medium containing 2% B27, 0.125 mM L-glutamine, and 1% penicillin/streptomycin twice per week. Neurons were used after 14 days.

Construct design. Expression plasmids for α3 subunit of Na^+/K^+-ATPase without or with pHluorin tag were generated by PCR amplification of cDNA sequence (rat ATP1A3) and insertion into pKR43 vector. The internal tag on the α subunit was made by site-directed mutagenesis between W307/L308. Superecliptic pHluorin (SEP) was subcloned with flanking flexible linkers (GGGGS) into the insertion site. All generated constructs were confirmed by sequencing analysis.

Membrane expression analysis. Confocal imaging was performed on an inverted Zeiss LSM 510 microscope with 63× (1.4 NA, oil) objectives. Cells were imaged during incubation in Krebs solution: 110 mM NaCl, 4 mM KCl, 1 mM NaH_2PO_4, 25 mM $NaHCO_3$, 1.5 mM $CaCl_2$, 1.2 mM $MgCl_2$, 10 mM glucose and 20 mM HEPES at pH 7.4 at 37°C. Confocal images were analyzed and quantified with the ImageJ software. For normalization of the intensity of fluorescence of our constructs tagged with SEP, we have used a commercial bead (InSpeck™ Green (505/515) Microscope Image Intensity Calibration Kit, 6 µm).

Localization of transfected ATP1A3 in membrane. Demonstration of plasma membrane expression transfected mutated and WT α3 in the plasma membrane (Fig 1A). Mutated and WT α3 were tagged in the extracellular domain with a pH-sensitive fluorescent probe (SEP) that had been inserted between transmembrane domains 3 and 4. The maximal fluorescence of SEP is observed at pH 7.4 and the signal will completely disappear at pH<5,0[31]. When the SEP tagged α3 isoforms were expressed in hippocampal neurons, we observed a bright signal for both WT and mutant α3 from what appeared to be the plasma membrane region (Fig. 1A and 1B). When extracellular pH was reduced from 7.4 to 4.2, the fluorescent signal rapidly decreased to less than 10% of the original value, and when pH was returned to 7.4, the signal was rapidly restored (Fig. 1C), proving that SEP is located extracellularly and indicating that the signal emerges from extracellular SEP. The strength of the fluorescent signal measured at pH 7.4 was normalized to an external fluorescent source and found to be similar for mutant and WT SEP tagged α3 (Fig. 1D). We conclude from these studies that mutant T613M and WT α3 isoforms are to a similar extent expressed in the plasma membrane.

Sodium imaging. Recordings were performed on primary hippocampal cell cultures after 16-21 day *in vitro* (DIV). The cells were transfected 2 days before imaging with *ATP1A3* WT or T613M together with mTurquoise2 (used to differentiate transfected from untransfected cells). Cells were loaded with the Na^+-sensing dye ANG2 (Asante NaTRIUM Green 2, excitation 517 nm) in Krebs solution: 110 NaCl mM, 4 KCl mM, 1 mM NaH_2PO_4, 25 mM $NaHCO_3$, 1.5 mM $CaCl_2$, 1.2 mM $MgCl_2$, 20 mM glucose, 1% BSA and 20 mM HEPES, pH 7.4 at 37°C for 25 minutes and then placed in a temperature controlled imaging chamber permitting the rapid exchange of pre-warmed perfusion solutions at 37°C. Fluorescence imaging was performed on inverted Zeiss Axiovert 200 with a 40× (1.4 NA, oil) objective equipped with an Andor iXon+ 897 EMCCD camera and with filter cubes selected for ANG2 and mTurquoise2. Cells were imaged during perfusion with aCSF (artificial cerebrospinal fluid): 125 mM NaCl, 26 mM $NaHCO_3$, 4 mM KCl, 1.2 $MgCl_2$, 1.25 mM NaH_2PO_4, 2 mM $CaCl_2$, 10 mM glucose and was continuously bubbled with carbogen (5% CO_2, 95% O_2) resulting in a pH of 7.4. The K^+-free solution (0 K^+) had the same composition, except that the NaCl and KCl concentrations were 129 mM and 0 mM, respectively. The NMDA solution had the same composition as aCSF with an addition of 20 μM NMDA. At the end of each experiment, the sample was perfused with Na^+ calibration solutions containing 0 mM and 15 mM Na^+ in the presence of 3 μM gramicidin, 10 μM monensin, and 1 mM ouabain. Na^+ calibration solutions contained $[Na^+ + K^+] = 165$ mM, 136 mM gluconate, 0.81 mM $MgSO_4$, 0.78 mM KH_2PO_4, 20 mM HEPES, 1.3 mM $CaCl_2$, pH adjusted to 7.2 with KOH. Images were analyzed and quantified with the ImageJ software. To correct for intensity variations between preparations, all values are expressed as the Na^+ calibrated ratio between transfected and untransfected cells recorded within the same sample.

Whole-cell patch clamp recordings of primary cultured neurons. Whole-cell patch-clamp recordings were performed on hippocampal cell cultures after 16–21 DIV. Cells were transfected 2 days prior to recordings with plasmids containing *ATP1A3* WT or T613M and EGFP for identification of transfected cells. Cultures were maintained with Krebs solution: 110 mM NaCl, 4 mM KCl, 1 mM NaH2PO4, 25 mM NaHCO3, 1.5 mM CaCl2, 1.2 mM MgCl2, 10 mM glucose and 20 mM HEPES at pH 7.4 at 37C. The internal pipette solution contained 120 mM potassium gluconate, 24 mM KCl, 4 mM NaCl, 4 mM MgCl2, 0.16 mM EGTA, 10 mM HEPES, 4 mM K2-ATP, pH 7.2 adjusted with KOH. Neurons were visualized using a fluorescence microscope and selected for whole-cell patch clamp recording if the cell soma was phase-bright and had been transfected. Recordings were performed using an Axopatch 200B amplifier (Molecular Devices) and pClamp software (version 8.2).

Animal model. We created a knock-in mouse model of T613M ATP1A3 using CRISPR/Cas technology. For our experiments, heterozygous and wild-type animals were crossed to get ratio WT and Het in a litter as 1:1. Genotyping was performed by using primers CGTGAACTTCACCACAGACAACCTT and GTGGAAGGGAGGAGTTGGAGGAGT and restriction step by using restrictase MlsI following PCR reaction. Mice were kept at a daily 12 h light/dark cycle. Experiments were performed with heterozygous mice and age-matched WT littermates. Age was approximately between 7-17 weeks. Mice were transferred to the behavior room one hour before testing.

Open field test. Spontaneous locomotor activity was recorded during an open field test. A mouse was placed in the center of the open field test apparatus and its velocity, total distance traveled, and time spent in the center area was recorded for 10 minutes.
These behaviors were analyzed with an automated video tracking system (Noldus Ethovision XT 11, Wageningen, Netherlands). For this test, both sexes of animals were used.

Elevated plus-maze. Anxiety was measured during the elevated plus-maze test. A mouse was placed in the center of the plus-maze apparatus where two arms of this apparatus were with walls and two were without. Total time spent in the open and closed zone was measured and analyzed with Noldus software. For this test, only males have been used.

Forced swim test. Depression-like behavior was measured by using a forced swim test. A mouse was placed in a plastic cylinder with warm (25±1ºC) water for 6 minutes and a total time of floating without active movement was analyzed with Noldus software. For this test, only males have been used.

Pole test. The postural control and motor coordination was tested using a pole test. A mouse was placed on the top of the vertical pole with the head to the top. Then the mouse turned and climbed onto the ground and time to turn and to reach a surface was calculated. This test contains 1 training trial (not calculated) and 5 testing trials and data presented as the mean of these 5 trials. For this test, both genders have been used.

Trigger studies. There are three triggers used in this study: immobilization for 1 hour under the light, standing in ice-cold water for 5 minutes, and alcohol intraperitoneal injection with 2.25 g/kg.

In vitro ventral root recordings from whole spinal cords. Isolated spinal cords were secured to the base of a recording chamber so that the ventral roots were accessible. The chamber was then continuously perfused with recording aCSF (~20mL/min; equilibrated with 95% oxygen 5% carbon dioxide gas). Plastic suction electrodes were then attached to either the first or the second ventral lumbar (L1/2) root on each side of the spinal cord to record flexor-mediated locomotor-related activity. Isolated spinal cords were allowed to recover for at least 45 minutes post-euthanisation before locomotor-related activity was recorded. Drug-induced locomotor-related activity was evoked via bath application of a dopamine (DA; 50uM), serotonin (5-HT; 10uM), and N-methyl-D-aspartic acid (NMDA; 5uM). Locomotor drugs were allowed to circulate until a stable locomotor rhythm was established. 'Stable' rhythms were classified as contralateral alternating ventral root bursts that were consistent in frequency, duration, and amplitude for at least 5 minutes, with rhythmic activity being analysed from the 10 minutes following this stabilisation. Signals were amplified and filtered (30-3000Hz; A-M Systems Differential AC Amplifier, Model 1700, Sequim, WA, USA) and then acquired at a sampling frequency of 3 kHz using a Digidata 1440A A/D board and AxoScope software (Molecular Devices, Sunnyvale, CA, USA). Ventral root data were analysed using Dataview software (v 10.3.0, courtesy of Dr. W. J. Heitler, University of St Andrews) before being imported into Excel and analysed. Rhythmic events were identified from rectified/integrated traces. Burst frequency and duration were measured from integrated traces while peak-to-peak amplitude was measured from corresponding raw traces. Amplitude was measured as a non-calibrated unit and is therefore presented as an arbitrary unit. Statistical analyses were conducted using GraphPad (Prism 9).

Whole-cell patch clamp recordings of motoneurons. Experiments were performed using heterozygous T613M mice and age-matched WT mice aged postnatal day (P)1-10. Neonatal mice were euthanised via cervical dislocation, decapitated, and eviscerated. Animals were secured to the base of a dissecting chamber filled with dissecting artificial cerebrospinal fluid (dissecting aCSF: 25mM NaCl, 188mM sucrose, 1.9mM KCl, 1.2mM NaH_2PO_4, 10mM $MgSO_4$, 1mM CaCl, 26mM $NaHCO_3$, 25mM glucose and 1.5mM kynurenic acid) equilibrated with 95% oxygen, 5% carbon dioxide at ~4°C. A vertebrectomy was performed, removing the

vertebrae covering the spinal cord. The cord was then isolated by separating the cord from the spinal roots with microscissors. Remaining dorsal or ventral roots or connective tissue were trimmed away if preparing slices. To produce spinal cord slices, the isolated cord was then set in 0.5% agar and immersed in a bath containing dissecting aCSF. A vibratome (Leica VT1200) was used to produce 300μm transverse slices of the lumbar region of the spinal cord. Lumbar slices were transferred to a chamber containing recovery aCSF (119mM NaCl, 1.9mM KCl, 1.2mM NaH2PO4, 10mM MgSO4, 1mM CaCl, 26mM NaHCO3, 20mM glucose and 1.5mM kynurenic acid), equilibrated with 95% oxygen, 5% carbon dioxide at ~34°C and allowed to recover for 30-45 minutes. Once recovered, the slices were transferred to a chamber containing recording aCSF (127mM NaCl, 3mM KCl, 2mM CaCl2, 1mM MgCl2, 26mM NaHCO3, 1.25mM NaH2PO4, 10mM glucose), equilibrated with 95% oxygen, 5% carbon dioxide at room temperature and allowed to equilibrate for approximately 30 further minutes before recording. For whole cord physiology experiments, the spinal cords were dissected in recording aCSF as opposed to dissecting aCSF before being transferred to a recording chamber containing circulating oxygenated recording aCSF ready for experimentation.

Spinal lumbar slices were placed in a recording chamber containing recording aCSF perfused at a continuous rate of ~1mL per minute. Whole-cell patch clamp recordings were made from motoneurons of the ventral horn. The intracellular solution for patch clamp recordings contained 14mM KMeSO4, 10mM NaCl, 1mM CaCl, 10mM HEPES, 1mM EGTA, 3mM Mg-ATP and 45mM Mg-GTP. Motoneurons were identified by morphology, passive properties, and location within the ventral horn. Lumbar motoneurons are in discrete ventromedial and ventrolateral pools and differ from interneurons in whole-cell capacitance and input resistance (Carlin, Jiang and Brownstone, 2000). Borosilicated glass microelectrodes (2.5-5 MΩ) containing intracellular solution were attached to the membrane of the neurons via a high resistance (>1 GΩ) seal. Suction was applied to rupture the patch of membrane within the seal, allowing the intracellular fluid within the glass microelectrode to become continuous with the inside of the cell. Signals were amplified and filtered with a MultiClamp 700B amplifier (Molecular Devices, Sunnyvale, CA, USA) and acquired at >10Hz using a Digidata 1440A A/D board and pClamp software (Molecular Devices, Sunnyvale, CA, USA). All cells used for experiments showed a resting potential between -50 and -80 mV. Firing output was measured in current-clamp mode with a bias current applied to all cells to maintain a consistent resting membrane potential of -60mV between recordings. Firing output was measured either as a single action potential acquired by injecting a 10ms supramaximal current pulse, a series of 1s square current pulses ranging from 50pA to 1250pA, or via a 10s square current pulse/pulse train (30Hz) applied at an amplitude of ~1.5x rheobase. Whole-cell patch clamp recordings were analysed using Clampfit software (Molecular Devices, Sunnyvale, CA, USA). For the analysis of single action potentials, 10 waveform samples were averaged into a single representative waveform. This representative trace was then used to calculate the firing threshold, amplitude, rise time, half-width, and mAHP measurements for each cell. Frequency-current (f-I) relationships were determined from a series of 1s square current pulses ranging from 50pA to 4000pA. Steady-state firing frequency (Hz) was determined from the last 500ms of the current pulse. Post-discharge activity denotes the activity of the cell after a period of prolonged firing (10s current pulse/train). Post-discharge activity area (mVs) is a combined measure of voltage change and recovery time. The area measurement represents the area under/over resting membrane potential starting at the cessation of firing and ending once resting membrane potential has been restored. Data are presented as mean ± standard error. In these data, the 'n' value represents the number of cells. All data collected were included in the statistical analysis.

Supplementary data

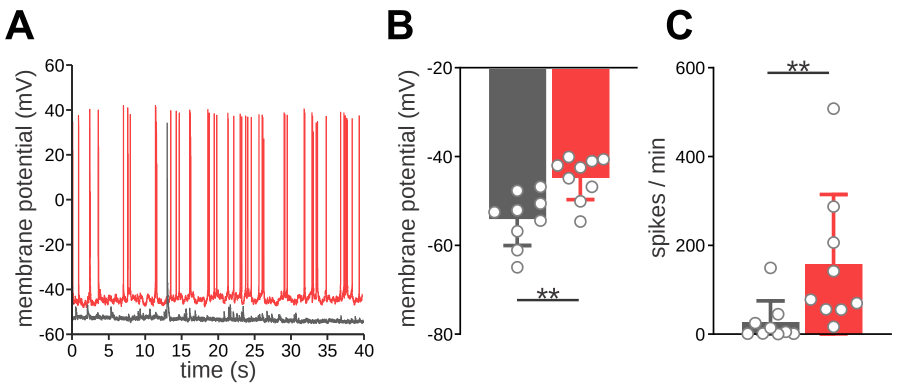

Supplementary Fig. 1: Increased excitability of hippocampal neurons expressing the T613M mutation

A. Examples of current-clamp recordings from hippocampal neurons expressing WT (black) or T613M (red) *ATP1A3*. B-C. Graphs showing the resting membrane potential (B) and the number of spikes per 1 min of gap-free recording (C) in hippocampal neurons expressing WT (black) or T613M (red) *ATP1A3*. n = 9 for each group. **, p<0.01 with Mann-Whitney rank test.

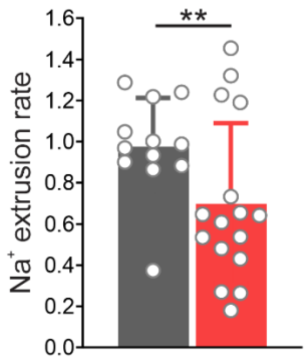

Supplementary Fig. 2: Impaired sodium extrusion in striatal neurons expressing the T613M mutation

The capacity to normalize an increase in intracellular sodium was also studied in neurons cultured from rat striatum expressing WT (black), or T613M (red) α3 Na^+/K^+-ATPase subunits (n=16 and 12, respectively). The time for normalization of an increase in intracellular sodium is also significantly longer in striatum neurons expressing the mutation.

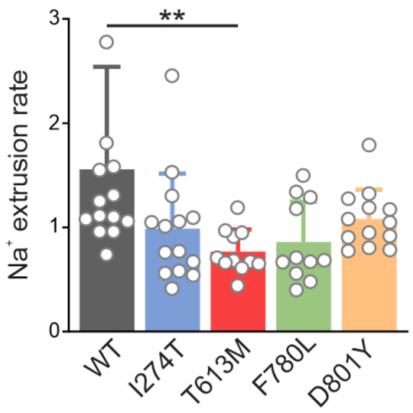

Supplementary Fig. 3: Intracellular sodium extrusion in four different RDP-associated ATP1A3 subunit mutations

The capacity to normalize an increase in intracellular sodium was studied in neurons cultured from rat striatum expressing 4 different mutations of *ATP1A3* that cause RDP. The time for normalization of an increase in intracellular sodium appeared to be delayed in all neurons expressing mutated α3 subunits but was only significant for the T613M mutation (WT: n = 14, I274T: n = 14, T613M: n = 11, F780L: n = 11; D801Y: n = 13).

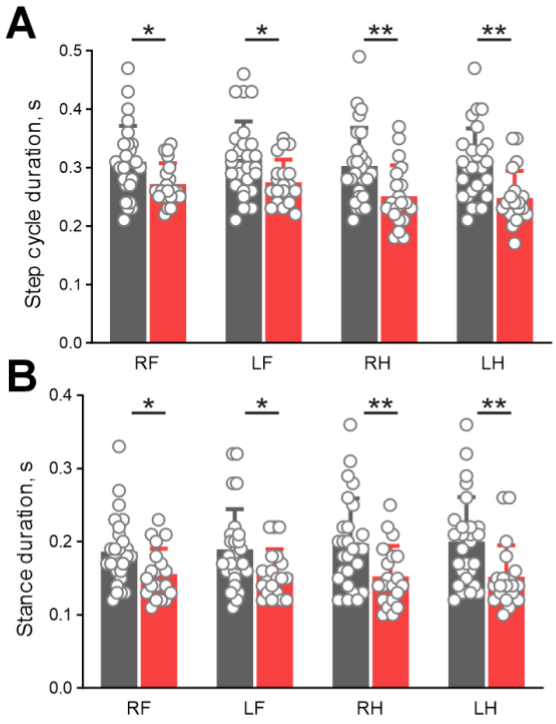

Supplementary Fig. 4: Forelimb and hindlimb kinematics

(A) T613M mice (n = 20) had a shorter step cycle duration across forelimbs and hindlimbs compared to WT mice (n = 27, Mann-Whitney Rank test).
(B) T613M mice (n = 20) had a shorter stance duration across forelimbs and hindlimbs compared to WT mice (n = 27, Mann-Whitney Rank test).

Supplementary information

		WT	T613M	p-value
Passive properties	Capacitance (pF)	110 ± 8.1	130 ± 6.2	0.11
	Input resistance (mΩ)	76 ± 6.2	77 ± 4.8	0.86
	Membrane potential (mV)	-59.11 ± 0.83	-62 ± 0.72	0.0081**
Single spike properties	Threshold (mV)	-45.54 ± 1.35	-45.66 ± 0.902	0.94
	Amplitude (mV)	75.27 ± 2.00	74.27 ± 1.42	0.69
	Rise time (ms)	0.96 ± 0.078	0.92 ± 0.042	0.63
	Half-width (ms)	0.90 ± 0.0754	0.93 ± 0.0483	0.8
	mAHP amplitude (mV)	-6.91 ± 1.03	-7.2 ± 0.39	0.8
	mAHP half-width (ms)	59.11 ± 8.99	61.27 ± 3.8	0.83
	mAHP duration (ms)	218.3 ± 27.42	218.6 ± 11.44	0.99
	mAHP area (mVs)	-526 ± 117.1	-538.8 ± 45.32	0.92
F-I relationship	Slope	0.036 ± 0.0034	0.035 ± 0.0021	0.73
	Max firing frequency (Hz)	31.35 ± 1.45	28.95 ± 1.081	0.19
	Min firing frequency (Hz)	10.76 ± 0.72	9.80 ± 0.82	0.38
	Repetitive firing threshold (pA)	313.3 ± 36.67	361.3 ± 36.57	0.36

Supplementary table 1. Electrophysiological properties of WT and T613M motoneurons.

Supplementary video 1. T613M animal with pronounced mobility deficits and tremor.

Supplementary video 2. T613M animal with gait abnormalities.

Supplementary video 3. T613M animal exhibiting hyperactivity.
